# Stereotactic implantation of diffusing alpha-emitters radiation therapy sources in the swine brain: a potential new focal therapy for brain tumors

**DOI:** 10.1007/s11060-024-04919-5

**Published:** 2025-01-02

**Authors:** Yigal Shoshan, Moshe J. Gomori, Lior Moss, Saleem Eben Bari, Nir Edery, Robert B. Den, Lior Arazi, Aron Popovtzer, Jon Feldman, Samuel Moscovici

**Affiliations:** 1 Department of Neurosurgery, Hadassah-Hebrew University Medical Center, Jerusalem, Israel; 2 Department of Radiology, Hadassah-Hebrew University Medical Center, Jerusalem, Israel; 3 Department of Pathology, Kimron Veterinary Institute, Bet Dagan, Israel; 4 Alpha Tau Medical, Jerusalem, Israel; 5 Unit of Nuclear Engineering, Faculty of Engineering Sciences, Ben-Gurion University of the Negev, Be’er-Sheva, Israel; 6 Sharett Institute of Oncology, Hebrew University-Hadassah Medical Center, Jerusalem, Israel

**Keywords:** Alpha DaRT, Stereotactic implantation, Minimally invasive, Focal therapy, Brain tumors

## Abstract

**Purpose:**

Diffusing alpha-emitters Radiation Therapy (“Alpha DaRT”) is a new cancer treatment modality that employs radium-224-loaded metal sources implanted in solid tumors to disperse alpha-emitting atoms within a therapeutic “kill-zone” of a few millimeters around each source. Preclinical studies have demonstrated tumor growth delay in various cancer types, including glioblastoma multiforme, and the method is used in clinical trials for patients with skin and head and neck cancer. This study aims to assess the safety and feasibility of implementing Alpha DaRT for brain tumor treatment in a large animal model.

**Methods:**

Alpha-DaRT sources were delivered via image-guided stereotactic implantation into both hemispheres of eight swine. 1–3 layers of radial deployment of 7 sources were delivered through a single penetration point into each hemisphere. A 90-day follow-up period included clinical evaluation, brain MRI, head CT, blood, CSF, urine, and feces sampling, and an analysis of source location over time. Brain tissue pathology was performed on termination.

**Results:**

Alpha-DaRT sources were reproducibly and efficiently delivered to the brain cortex and subcortex. No unexpected abnormalities were detected in blood or CSF samples. MRI and CT scans revealed no evidence of major bleeding or infection. Measurements of ^212^Pb in blood and CSF exhibited the expected exponential decay from day 7 to day 14 post-source implantation. Minimal spatial and temporal movements of the sources were noted. Histopathological analysis demonstrated locally confined findings in brain parenchyma in a very close proximity to the sources.

**Conclusion:**

Alpha**-**DaRT sources can be safely delivered into a large animal brain using image-guided stereotactic implantation. These findings support further exploration of Alpha DaRT as a potential treatment modality for brain tumors.

**Supplementary Information:**

The online version contains supplementary material available at 10.1007/s11060-024-04919-5.

## Introduction

Primary brain tumors, specifically high-grade gliomas, and metastatic brain cancer, stand as the most prevalent malignant brain tumors [1–3]. These conditions carry significant morbidity and mortality rates [4]. The standard approach to managing these tumors typically entails a multifaceted treatment regimen, including surgical intervention, radiation therapy, and chemotherapy [5]. Despite dedicated efforts, recurrences remain a persistent challenge, necessitating further interventions such as stereotactic irradiation, ablative procedures, and targeted medical therapies.

Alpha particles are a type of high linear energy transfer (LET) radiation, which deposit a large amount of energy (few MeV) over a few tens of micrometers, leading to significantly more severe and complex DNA damage than low-LET treatments (based on photons and electrons), as well as proton beams [6, 7]. The high relative biological effectiveness (RBE) of alpha radiation presents a compelling avenue for treating otherwise incurable brain tumors, particularly recurrent tumors in which normal tissue has already been damaged. Compared to currently available therapies such as external beam radiosurgery with gamma rays (Gamma Knife), X-rays (X-Knife), or external proton radiation, in this approach radiation is delivered directly into the tumor and the sharp dose fall-off reduces the risk of radiation necrosis and spares normal surrounding tissue. This is particularly important for patients who have previously undergone external radiation therapy, with is already underlying damage and radionecrosis to normal tissue.

In addition, while gamma or beta radiation require a reservoir of oxygen to damage the DNA and may be more efficient in killing fast growing cells, cancer cell death induced by alpha particles is nearly independent on oxygen or cell cycle phase [8, 9]. Recurrent tumors tend to be more hypoxic with areas of both acute and chronic hypoxia [10], and to origin from quiescent slow-growing cancer stems cells [11] thus rendering gamma and beta radiation less efficacious. Nevertheless, the clinical use of alpha radiation has so far been limited due to the short range of alpha particles in tissue (< 0.1 mm). While many schemes for targeted alpha therapy are under development and in clinical trials [12–14], they are generally considered to be more suitable for the treatment of single-cell or micrometastatic disease rather than solid tumors. At present, the only approved alpha-particle-based therapy is the treatment of bone metastases in castration-resistant prostate cancer using intravenous injection of ^223^RaCl_2_ [15].

Alpha-DaRT is a new treatment modality which enables effective use of alpha particles against solid tumors. The method relies on the use of implantable metallic sources, each carrying a few µCi radium-224 (^224^Ra, 3.63-d half-life). Alpha-DaRT sources offer a potential solution to the short-range problem of alpha-particles as they are designed to maintain the cell-killing potency of alpha particles but increase the spread of the dose to clinically meaningful margins (few mm around the source) using the diffusion of alpha-emitting atoms inside the tissue [16], allowing their use as focal treatment. Alpha DaRT demonstrated efficacy in preclinical human- or murine- tumor-derived models including breast, prostate, pancreas, skin, lung, colon, and glioblastoma multiforme [16–23]. In a first-in-human clinical trial, Alpha DaRT reached a 78.6% (22/28) complete response rate in skin, head and neck cancers [24] and a second trial in skin cancers demonstrated a 100% (10/10) complete response rate [25].

Given the unmet medical need for patients with brain tumors, and the potential benefit of Alpha DaRT, this study was conducted to determine the feasibility of deploying Alpha-DaRT sources in a predefined configuration using a minimally invasive approach, and to assess safety aspects of the treatment on a healthy porcine brain.

## Methods

### Animals

The study was conducted at the animal facility of Lahav Research Institute (LRI), Kibbutz Lahav, Israel. All animal experiments were carried out in accordance with the government and institution guidelines and regulations and approved by the Israeli National Animal Care and Use Committee (Ethics approval NPC-La - IL − 2212–359-4). Eight 61.6–98.9 kg, 4–5 months old female domestic swine participated in the study.

### Alpha-DaRT sources

Titanium-based Alpha-DaRT sources were made of Grade 2 titanium cylinders (0.37 mm in diameter, 10-mm long) that were either inert or loaded with ^224^Ra atoms (activity of 3 µCi, i.e.,0.11 MBq) by an electrostatic collection process similar to that previously described [16]. The sources in this study were coated with a 1-µm-thick polydimethylsiloxane polymeric layer (Nusil, MED2-4213). The ^220^Rn desorption probability (the probability that a ^220^Rn atom is emitted from the source following a decay of ^224^Ra) was about 40–45%. Several sources were inserted into the brain hemispheres using an Alpha-DaRT radial applicator according to a predetermined configuration (Fig. S1A-B) creating a radiation dose field calculated based on a dedicated physics model [26, 27].

### Alpha DaRT radial applicator

A dedicated applicator, designed for the implantation of permanent sources in the brain, guided by a standard brain biopsy needle and its accessories, was developed (Fig. S1A). The applicator allows the insertion of multiple Alpha-DaRT sources into the brain tissue in a configuration of several layers of 7 sources in an umbrella-like radial deployment (Fig. S1B). The procedure consists of deploying a first umbrella-like layer at a desired level, followed by needle retraction such that the next level is deployed above the first, and so on. This configuration provides effective coverage of a predefined target volume in terms of the spatial spread of alpha-emitters.

### Image-guided stereotactic implantation procedures

Seven to ten days prior to source implantation, three metallic bone anchors were implanted in the skull (two on the zygomatic arch and another one on the parietal bone), the skin was sutured on top of each anchor, and a computed tomography (CT) scan of the head was performed under general anesthesia (see Supplementary Information). The placement of the anchors was necessary to recapitulate a stereotactic approach in the porcine model. CT DICOM files were used to define a virtual tumor (target) inside the brain and an entry point by the surgeon. The target contour and entry point were then used to produce a guiding template frame (Synergy, Israel), which was printed and sterilized before use (Fig. S1C-1G).

**Fig. 1:**
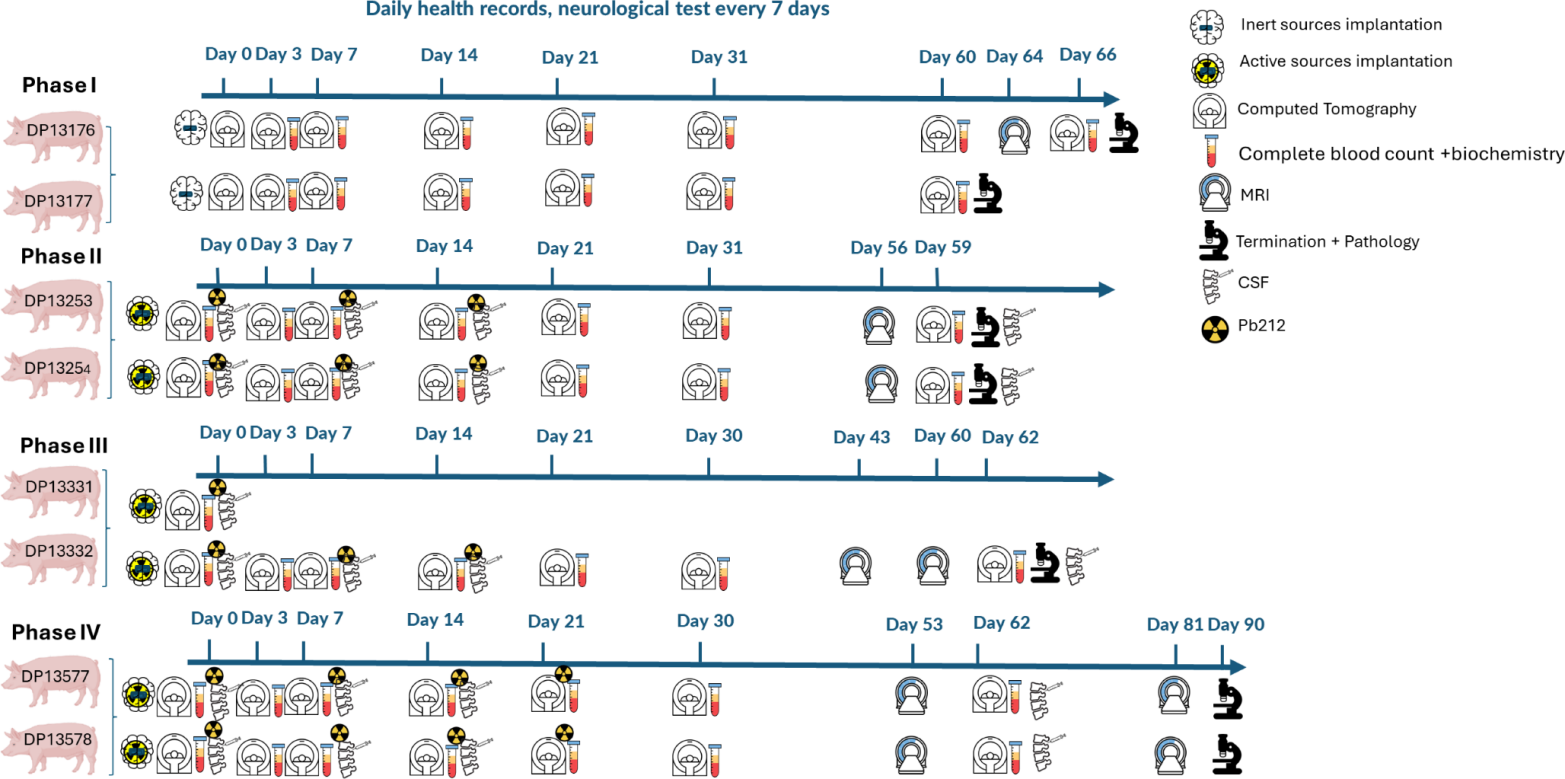
Experimental design. The study was conducted in four phases, two swine in each phase. In the first phase, animals were implanted with inert sources, and in phases 2–4 with active sources. Follow-up included daily health records, neurological evaluation, CT scans, MRI and hematology, and biochemistry. For active phases, CSF was collected and ^212^Pb levels were evaluated. By the end of the follow-up period, the animal’s brain was subjected to gross pathology and histopathology. CT scans were used for movement analysis. The following are exceptions from the design: (1) For animals DP13577 and DP13578 CSF was sampled on day 62 but sampling did not allow analysis. (2) for animals DP13253, DP13254, DP13332 on day 7 CSF was sufficient only for ^212^Pb test but not for the CSF test. (3) Inert blood test included CBC for both swine on days 3, 7, 14, 21, 31, and 60. Biochemistry was taken for swine DP13176 on days 3 and 10 and for swine DP13177 only on day 3

On treatment day (Fig. 1), the animal was anesthetized, and the skin over the skull and over the anchor points underwent thorough cleaning and scrubbing. Anchors were exposed, and the template frame was adjusted to fit them precisely. The template frame was then fixed to the anchors with screws. A 2.5-mm twist drill was passed through a built-in tunnel of the template frame to make a hole through the skin and the bone in one pass. The biopsy needle, configured with a stylet and cartridge, was inserted into the same tunnel toward the target volume. The radial cartridge contained 7 sources, and in the case of active sources, it was washed before the procedure with saline. The sources were inserted into the brain’s cerebral hemispheres using the Alpha-DaRT radial applicator as described above.

Since the swine brain is smaller than the human brain (150 vs. 1500 cc, respectively), a preliminary study was performed to determine the exact target location of the sources in a swine brain. The target location was defined as the cortex or subcortex of the parietal lobe, avoiding the olfactory bulbs, thalamus, and brain stem. The implants were inserted through entry points in the parietal bone, near the frontal-parietal suture line. These entry points were situated 17 mm from each other and were equidistant from both the midline suture and the frontal-parietal suture line.

For imaging procedures (intraoperative fluoroscopy, postoperative CT, and MRI), follow-up procedures (animal clinical and neurological examination, CSF and blood tests of ^212^Pb and external dose-rate measurements, pathology and histopathology) and spatial-temporal source location analysis, please see Supplementary Information.

### Data availability

The data will be made available upon reasonable request.

## Results

### Feasibility of Alpha-DaRT source deployment according to a treatment plan

**Fig. 2:**
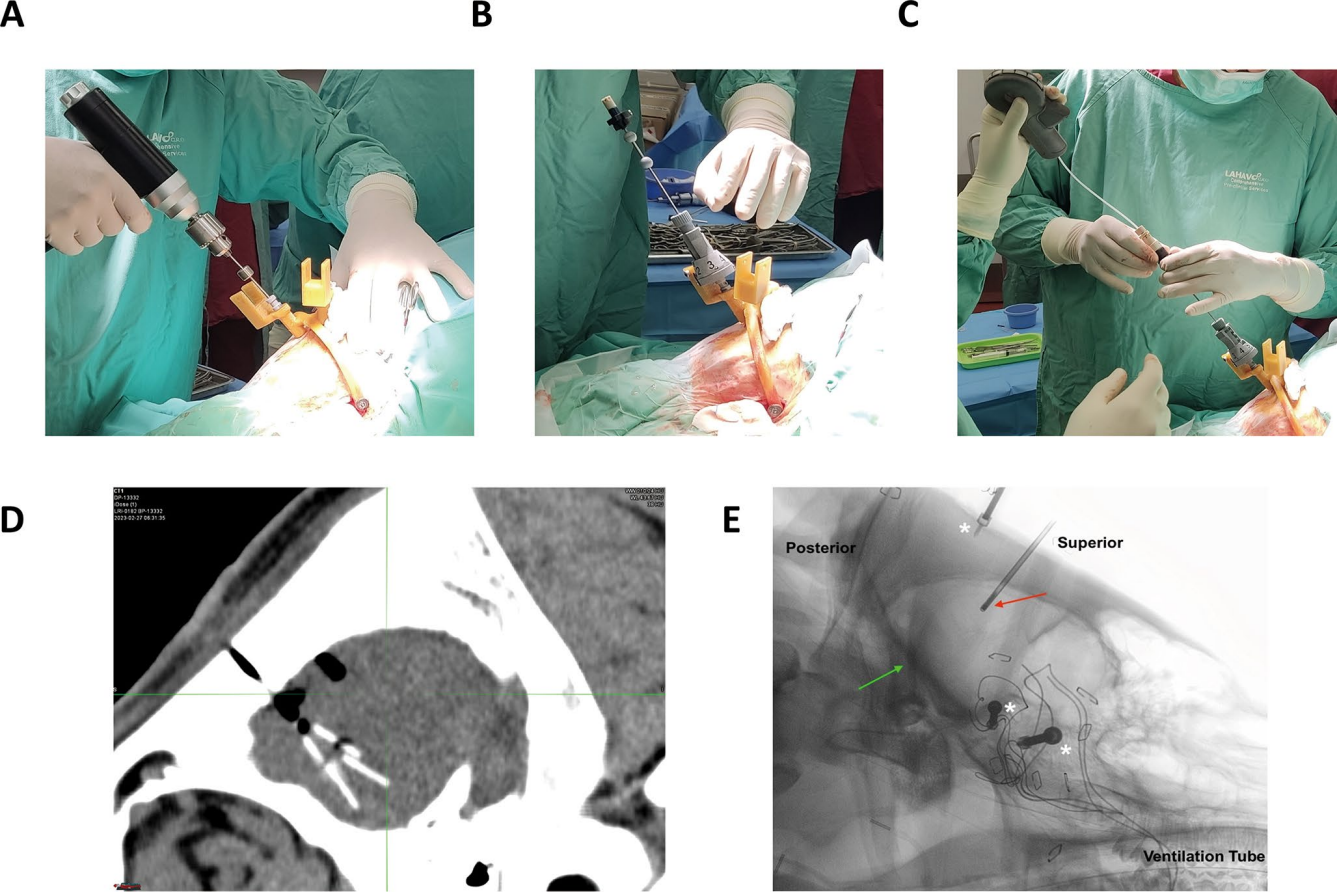
Representative images of Alpha-DaRT sources implantation to the swine brain. **(A)** After assembling the template-frame on the swine ‘s head, holes were drilled to access the brain. **(B)** The radial applicator was assembled on the template-frame and a biopsy needle was fixed on the rotation mechanism. **(C)** The Radial Cartridge loaded with sources was inserted into the biopsy needle and the sources were deployed one by one at the planned location in an umbrella-like configuration using the handle. **(D)** the umbrella-like configuration of Alpha-DaRT sources is presented under the entry point (drilling hole). **(E)** Intraoperative fluoroscopy of anesthetized swine for applicator needle localization verification prior to source implantation. Red arrow – applicator’s needle tip. Green arrow – base of the skull. White asterisk - three (3) metal screws of the customized stereotactic frame applied to the animal skull

A total of 206 sources were implanted in four phases, with two swine in each phase (see Table S1, Fig. 1). In the first phase, the sources used were not radioactive (inert sources), while in the subsequent three phases, all sources were active. The radial applicator was easy to use and placed multiple sources in a relatively short time (minutes) according to the predetermined configuration (Fig. 2). The duration of the entire procedure did not exceed one hour per animal, including anchor placement and implantation of all sources in both hemispheres (up to 42 sources per animal).

**Fig. 3:**
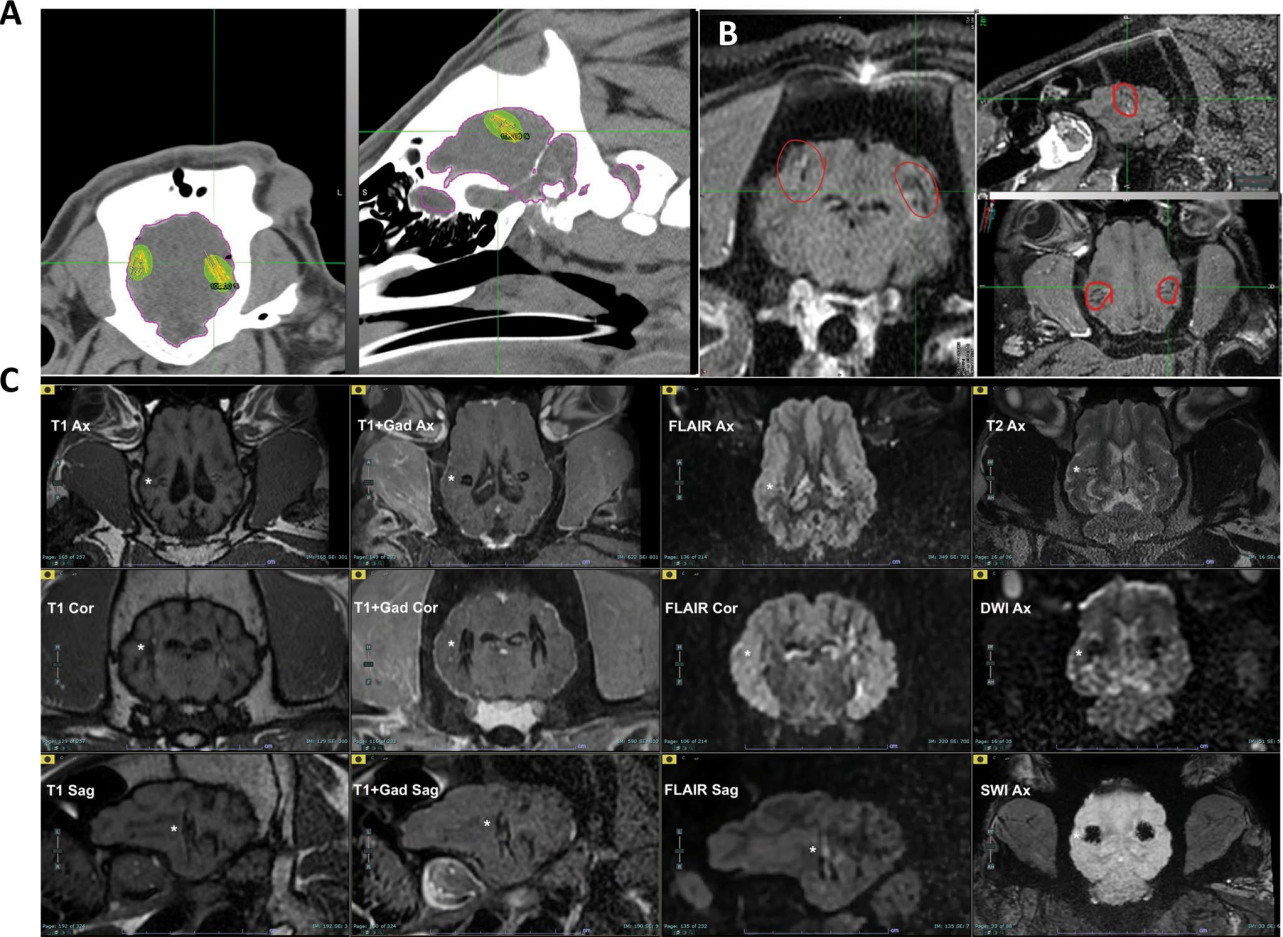
CT and MR scans of Alpha-DaRT sources in the swine’s brain. **(A)** Two-planar CT images. In each image, the virtual sources (i.e., the sources as represented in the treatment plan software) are shown as green marks, while the estimated region subject to an alpha dose of > **20** Gy they create is shown as a green-filled contour. The brain contour is represented in magenta. **(B)** Three-planar MR images for the same animal as A. The sources are shown as black stains and are marked by red circles. **(C)** Three-planar MR Image data sets with various sequences commonly utilized for brain tumors (T1W ± Gadolinium, FLAIR, T2W, DWI, and SWI) of animal DP13253 brain. Implant location-bilateral parietal. Mild intra-implant microhemorrhages left greater than right. No evidence of infection or inflammation. Minimal intra-implant tissue edema bilaterally. White asterisk – near right side implant location

Following the insertion of the sources in 1–3 layers of 7 sources (unless mentioned otherwise) per layer to the left or right hemispheres, animals were monitored for a period of up to 90 days. During the follow-up period, CT and MRI scans (Fig. 3), blood, CSF, urine, and feces samples were collected, and clinical and neurological evaluations were performed.

Due to the close proximity of the biopsy needle to the skull during the deployment of the sources in one animal (DP13577), one source did not come out of the cartridge (see Table S1).

### Movement of sources in the follow-up period

Analysis of source movement was done relative to the 3D source enclosure, defined as the minimal convex surface that encapsulates all sources together with a 3.6-mm margin, corresponding to registration errors and errors in identifying the source coordinates (see more details in the Supplementary Information). Out of a total of 206 sources, only one source partially protruded out of the 3D source enclosure with the maximal distance of 0.6 mm (Table 1, Fig. S3). In the first animal operated on in the study (swine DP13176), one source was unintentionally inserted into the middle part of the right ventricle due to the pig ventricles not being clearly visible on CT. This source moved through the CSF-filled ventricle anteriorly to the frontal horn between days 14 and 21. The target location was refined for subsequent insertions. This source was excluded from the movement analysis. Notably, by comparing days 0–60 and days 0–66 of animal DP13176 it can be shown that the MRI performed on day 64 did not affect the movement level.

**Table 1:** Spatial-temporal localization analysis

Swine #	Number of Outliers	Number of entire outliers	Outliers max distance (mm)	Total error (mm)	First follow-up day	Last follow-up day
13,176	0	0	0	5.50	0	60
13,176	0	0	0	3.56	0	66
13,176	0	0	0	2.75	60	66
13,177	0	0	0	3.79	0	60
13,253	0	0	0	4.88	0	59
13,254	1	0	0.61	2.82	0	59
13,331	0	0	0	3.78	0	1
13,332	0	0	0	2.85	0	62
13,577	0	0	0	2.44	0	62
13,578	0	0	0	3.89	0	62
			**Mean total error**	**3.62**		

Outliers” and “Entire outliers” denote sources that were partially or entirely out of the therapeutic envelop, respectively; “Distance” denote the distance of protruding sources from the marginalized dose. “Total error” is expressed by the square root of (registration error ^2 + identification error^2)

### Clinical and neurological evaluation during the follow-up period

All clinical evaluations including neurological evaluations were normal except for one animal (DP13254) that exhibited temporary neurological deficiencies, starting from day 7. The neurological symptoms were mild front legs ataxia, mild head tilt and circling to the right, and possibly vision impairment. These symptoms, except for the suspected vision impairment, gradually improved and were resolved by day 23. These clinical signs were mild and did not prevent the animal from eating. Therefore, it was not prematurely euthanized and completed the entire trial. Notably, in this animal, the sources were placed at a deeper location than planned in the left hemisphere as can be seen in Fig. S4. No treatment-related findings were observed in follow-up measurements.

### CSF, hematology, and chemistry

No abnormal findings were found in the blood tests. CSF, glucose, and lactate values were normal. Total protein values were elevated in all animals on day 14, as may be expected from the tissue damage following source insertion. In animal DP13253, the high values (179 mg/dl) increased steadily throughout the trial. Animal DP13332 had elevated levels of total protein in CSF that normalized by day 60. In one animal (DP13254) the total protein values were very high (416 mg/dl, peak on day 14), and on day 60 the levels were still high but lower than on day 14. This animal had the highest number of sources implanted. For the other two swine, no CSF samples were available on termination day. Of note, there was no correlation between clinical symptoms and total protein levels.

### Postoperative MRI analysis

All MR Image data sets were reviewed by a senior neuro-radiologist (JMG). MRI scans did not show any evidence of infection or inflammation. All active animals had mild peri- or intra-implant-zone edema. The local damage to the tissue surrounding the sources was demonstrated (minimal peri- /intra-implant tissue reaction) without any non-adjacent tissue reaction or other clinically significant abnormal findings. All active animals had mild intra-implant microhemorrhage. One animal (DP13578) had one macroscopic peri-implant hemorrhage on day 53, which had no clinical significance based on the animal’s neurological examination. It was not observed on MRI day 81 as it was probably absorbed. The clinical follow-up of this animal was extended to day 90 with no relevant findings.

### ^212^Pb and geiger counter measurements

^212^Pb specific activity (corrected from the measurement time to the sample collection time) on days 7–8 was 1.5-5.0 Bq/ml in blood, 0.7–14.8 Bq/ml in CSF, 1.1–2.9 Bq/ml in urine, and 0.1-3.0 Bq/g in feces. The ratio between the blood, CSF, and urine-specific activities on day 14–15 and day 7–8 was consistent, within error, with the expected decay of ^224^Ra (with which ^212^Pb is in secular equilibrium from ~ 2 days post-treatment and onward). No feces samples were taken on days 14–15. When normalized by the total ^224^Ra activity on the Alpha-DaRT sources (calculated for the time of sample collection), the specific ^212^Pb activity per µCi ^224^Ra on day 7–8 was (mean ± std) 0.14 ± 0.04 Bq/ml/µCi for blood, 0.16 ± 0.16 Bq/ml/µCi for CSF, 0.11 ± 0.05 Bq/ml/µCi for urine, and 0.04 ± 0.04 Bq/g/µCi for feces. Notably, the normalized specific activity recorded in blood is > 10 times smaller than observed in Alpha-DaRT-treated patients (with skin and head and neck tumors) [24]. This may indicate possible reduced clearance of ^212^Pb by the blood due to the blood-brain barrier. A gross estimate for the alpha-particle absorbed dose to the swine brain (outside of the treatment region) is described in the Supplementary Information. For the largest treatment in this study, consisting of 126 µCi ^224^Ra, it is ~ 4 × 10^− 4^ Gy, ~ 30,000 timessmaller than the estimated tolerance alpha dose of 12 Gy [28].

The external dose rate was measured at 0–30 cm away from the swine’s head in two swine (along with a comparative background measurement) during the first 5 days from sources insertion. The values (e.g., ~ 0.01 mGy/h at 30 cm for 126 µCi ^224^Ra) were similar to those observed in skin patients at 30 cm and consistent with exposure estimates using the MicroShield code [13].

### Pathology and histopathology

**Fig. 4:**
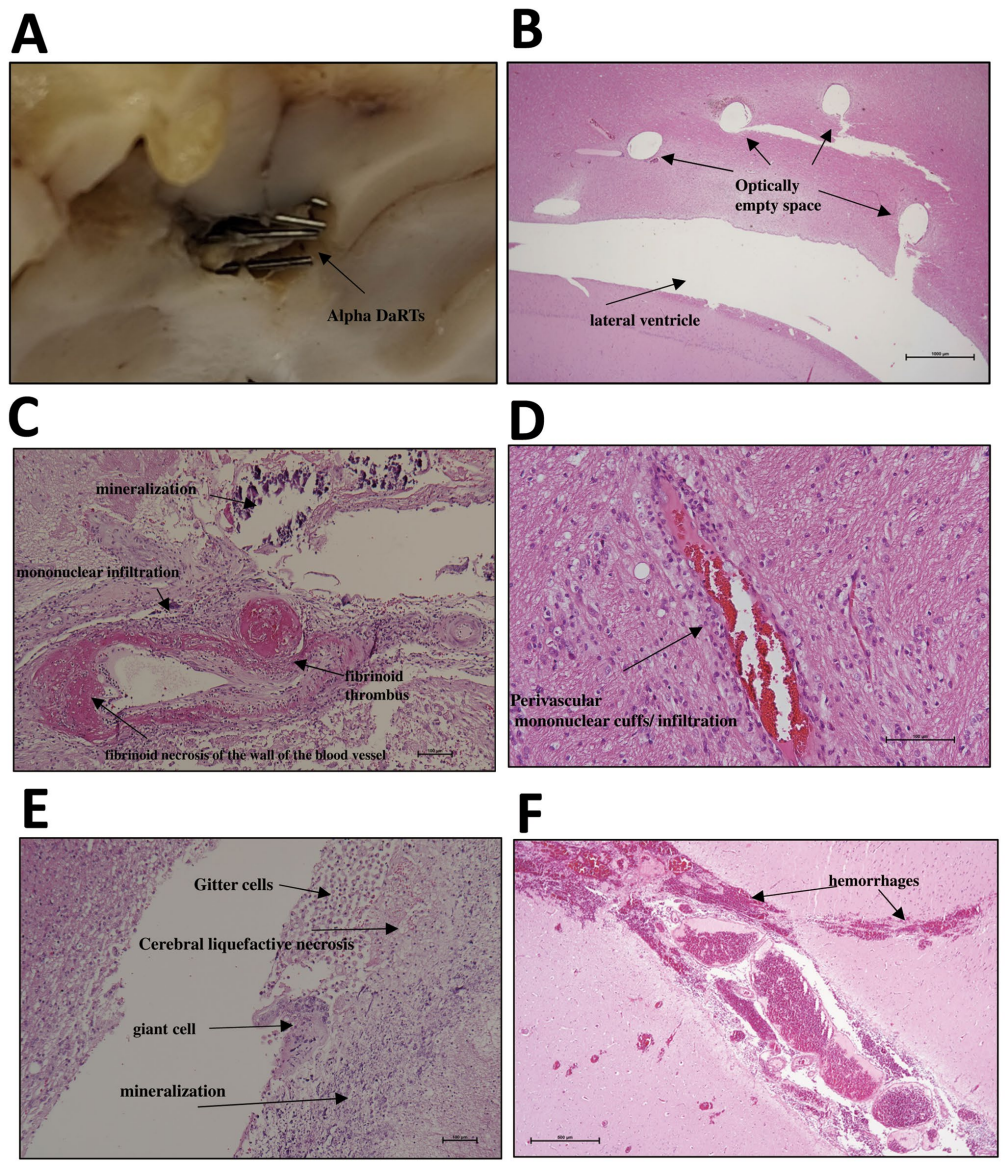
Representative images of the swine’s brain pathology at the end of the follow-up period, post Alpha-DaRT insertion. **A**. Gross pathology of a cluster of Alpha**-**DaRT sources in cerebral cortex, swine. **B**. Five optically empty spaces in the cerebrum adjacent to the lateral ventricle. **C**. Optically empty spaces (circular & longitudinal) surrounded by mineralization, fibrin, foreign body material (bone dust and fragments & keratin), fibrinoid necrosis of the parenchyma and the wall of blood vessels and intravascular fibrinous thrombi. **D**. Mononuclear perivascular cuffs in cerebral parenchyma. **E**. At the margins of the optically empty space the histopathologic findings include neutrophils and eosinophils inflammatory infiltrate, reactive endothelium, giant cells, liquefactive necrosis, edema in the neuropil and Gitter cells. **F**. Meningeal and parenchymal cerebral hemorrhages

Macroscopically, in all swine, the Alpha-DaRT sources were located within the cortex and subcortex cerebral regions (Fig. 4A). None of the brains presented evidence of gross tissue necrosis. In animal DP13176 the histopathological analyses showed that one Alpha-DaRT source was located at the frontal horn of the right ventricle. In swine DP13177 several Alpha-DaRT sources penetrated the caudate nucleus.

The tissue findings were consistent with moderate to severe, focal-extensive, meningoencephalitis with liquefactive necrosis. In all the sections, the histopathological findings were locally confined adjacent or around the Alpha-DaRT sources or cluster of Alpha-DaRT sources. Namely, no abnormal findings were observed at distant sites (not beyond approximately 3–8 mm from the source, according to gross pathology observations) in active sources compared to inert sources.

In all animals, the histopathological findings were localized with varying degrees of severity (Fig. 4B and F). In a few sections, histopathological findings included minimal vacuolization (possible artifact), hemosiderin-laden macrophages, reactive meningeal cells, and “red neurons” (suspected as neuronal necrosis).

## Discussion

In this study, we demonstrated the feasibility and safety of image-guided stereotactic locally delivered alpha-emitters, using the investigational device Alpha DaRT, to both the cortex and subcortex brain tissues in a healthy swine. The deployment and implantation of the necessary sources proved to be highly efficient. This efficiency was achieved through the utilization of a dedicated applicator, facilitating the delivery of seven sources at each level and across multiple levels in a single penetration pass, using a standard brain biopsy needle. It is noteworthy that this method can potentially be seamlessly adapted into a neuro-navigation platform for the treatment of human subjects. Analysis of the source’s spatial-temporal localization indicated minimal movement of Alpha-DaRT sources within the target volume.

Alpha DaRT proposes a method for the delivery of high-LET radiation to primary brain tumors and metastases with several potential benefits. Alpha-DaRT sources, loaded with low activity (a few microcurie) ^224^Ra, should be able to deliver a more conformal dose of radiation with a higher relative biological effectiveness to the tumor and less dose to normal tissue. This is especially critical in the population of patients who have received previous stereotactic radiosurgery and/or radiotherapy, thus making them more susceptible to radiotherapy-related side effects, specifically radionecrosis. Indeed, the study showed that in contrast to other radiotherapies, Alpha-DaRT sources do not lead to any significant distant effect and preserve the brain tissue outside of the ablative area. As to the possibility of radiation dose to brain tissue due to the presence of ^212^Pb activity in the blood or CSF, our analysis indicates that under realistic overall ^224^Ra activities, such dose levels are expected to be negligible.

During the Alpha-DaRT-based treatment, several sources are inserted into the tumor according to a pre-determined treatment plan. The delivery ensures that an adequate tumoricidal dose will be delivered to the tumor. Using a pre-procedure MRI, the tumor with margin can be delineated and the treatment plan can be overlayed onto the MRI imaging, thus allowing the neurosurgeon to direct the deployment accordingly.

The study highlighted that the location of the sources’ placement is critical when considering the treatment and should be limited to brain parenchyma. In addition, the location of the biopsy needle should avoid proximity to the calvarium and the skull-base to allow a free departure of the sources from the applicator. The transient increase in CSF protein levels indicates that the Alpha-DaRT procedure induced damage to the target (normal brain parenchyma and local blood brain barrier).

As the primary goal of the proposed technology is to treat brain tumors, the histological and radiological findings observed in this study support the aim of tumor eradication without inducing damage to adjacent or distant tissue. Histopathological findings also identified local immune cells infiltration in the brain, indicating a potential immune reaction to the treatment. Additional exploration is required to examine the nature of this response and to determine whether it may support a strategy of combining Alpha DaRT with immunotherapy, which has been shown to be no worse than Avastin in the setting of recurrent disease [29].

### Limitations

This study primarily aimed to evaluate the feasibility and safety of Alpha DaRT implantation into the healthy brains of large animals, rather than investigating a spontaneous brain tumor model in such animals. The extent of tumor volume coverage achieved by the implantation technique was not examined, underscoring the need for further investigation. Implanting radiation sources into healthy brain tissue may not fully represent the potential hemorrhagic complications associated with implantation into brain tumors. Additionally, while the 90-day follow-up period provided insight into early effects, it may not adequately capture long-term outcomes, such as radiation necrosis, fibrosis, or delayed complications.

### Conclusion

The current preclinical study demonstrates the feasibility and safety of image-guided stereotactic delivery of Alpha-DaRT sources through a minimally invasive approach. In addition, it confirmed locally confined tissue damage around Alpha DaRT sources, sparing healthy brain tissue. These successful findings provide the necessary support to initiate clinical trials investigating Alpha DaRT as a novel potential focal therapy modality for brain tumors in patients with recurrent brain tumors.

## Electronic supplementary material

Below is the link to the electronic supplementary material.

Supplementary Material 1

## Data Availability

No datasets were generated or analysed during the current study.

## References

[1] Ostrom QT, Gittleman H, Xu J, Kromer C, Wolinsky Y, Kruchko C, Barnholtz-Sloan JS (2016) CBTRUS Statistical Report: primary brain and other Central Nervous System tumors diagnosed in the United States in 2009–2013. Neuro Oncol 18:v1–v75. 10.1093/neuonc/now207 28475809 10.1093/neuonc/now207 PMC8483569

[2] Lamba N, Wen PY, Aizer AA (2021) Epidemiology of brain metastases and leptomeningeal disease. Neuro Oncol 23:1447–1456. 10.1093/neuonc/noab101 33908612 10.1093/neuonc/noab101 PMC8408881

[3] Ostrom QT, Wright CH, Barnholtz-Sloan JS (2018) Brain metastases: epidemiology. Handb Clin Neurol 149:27–42. 10.1016/B978-0-12-811161-1.00002-5 29307358 10.1016/B978-0-12-811161-1.00002-5

[4] Heron M (2019) Deaths: leading causes for 2017. Natl Vital Stat Rep 68:1–77 32501203

[5] Stupp R, Mason WP, van den Bent MJ, Weller M, Fisher B, Taphoorn MJ, Belanger K, Brandes AA, Marosi C, Bogdahn U, Curschmann J, Janzer RC, Ludwin SK, Gorlia T, Allgeier A, Lacombe D, Cairncross JG, Eisenhauer E, Mirimanoff RO, European Organisation for R, Treatment of Cancer Brain T, Radiotherapy G, National Cancer Institute of Canada Clinical Trials G (2005) Radiotherapy plus concomitant and adjuvant temozolomide for glioblastoma. N Engl J Med 352: 987–996 10.1056/NEJMoa043330 10.1056/NEJMoa043330 15758009

[6] Thompson JM, Elliott A, D’Abrantes S, Sawakuchi GO, Hill MA (2019) Tracking down Alpha-Particles: the design, Characterisation and Testing of a shallow-angled alpha-particle irradiator. Radiat Prot Dosimetry 183:264–269. 10.1093/rpd/ncy300 30726978 10.1093/rpd/ncy300 PMC6525335

[7] Malouff TD, Mahajan A, Krishnan S, Beltran C, Seneviratne DS, Trifiletti DM (2020) Carbon Ion Therapy: a modern review of an Emerging Technology. Front Oncol 10:82. 10.3389/fonc.2020.00082 32117737 10.3389/fonc.2020.00082 PMC7010911

[8] Barendsen GW, Koot CJ, Van Kersen GR, Bewley DK, Field SB, Parnell CJ (1966) The effect of oxygen on impairment of the proliferative capacity of human cells in culture by ionizing radiations of different LET. Int J Radiat Biol Relat Stud Phys Chem Med 10:317–327. 10.1080/09553006614550421 5297012 10.1080/09553006614550421

[9] Hall EJ, Gross W, Dvorak RF, Kellerer AM, Rossi HH (1972) Survival curves and age response functions for Chinese hamster cells exposed to x-rays or high LET alpha-particles. Radiat Res 52:88–98 4672846

[10] Walsh JC, Lebedev A, Aten E, Madsen K, Marciano L, Kolb HC (2014) The clinical importance of assessing tumor hypoxia: relationship of tumor hypoxia to prognosis and therapeutic opportunities. Antioxid Redox Signal 21:1516–1554. 10.1089/ars.2013.5378 24512032 10.1089/ars.2013.5378 PMC4159937

[11] Basu S, Dong Y, Kumar R, Jeter C, Tang DG (2022) Slow-cycling (dormant) cancer cells in therapy resistance, cancer relapse and metastasis. Semin Cancer Biol 78:90–103. 10.1016/j.semcancer.2021.04.021 33979674 10.1016/j.semcancer.2021.04.021 PMC8576068

[12] McDevitt MR, Sgouros G, Sofou S (2018) Targeted and nontargeted alpha-particle therapies. Annu Rev Biomed Eng 20:73–93. 10.1146/annurev-bioeng-062117-120931 29345977 10.1146/annurev-bioeng-062117-120931 PMC5988956

[13] Working TAT, Parker G, Lewington C, Shore V, Kratochwil N, Levy C, Linden M, Noordzij O, Park W, Saad J F (2018) Targeted alpha therapy, an emerging class of Cancer agents: a review. JAMA Oncol 4:1765–1772. 10.1001/jamaoncol.2018.4044 30326033 10.1001/jamaoncol.2018.4044

[14] Tafreshi NK, Doligalski ML, Tichacek CJ, Pandya DN, Budzevich MM, El-Haddad G, Khushalani NI, Moros EG, McLaughlin ML, Wadas TJ, Morse DL (2019) Development of targeted alpha particle therapy for solid tumors. Molecules 24. 10.3390/molecules24234314 10.3390/molecules24234314 PMC6930656 31779154

[15] Shirley M, McCormack PL (2014) Radium-223 dichloride: a review of its use in patients with castration-resistant prostate cancer with symptomatic bone metastases. Drugs 74:579–586. 10.1007/s40265-014-0198-4 24610703 10.1007/s40265-014-0198-4

[16] Arazi L, Cooks T, Schmidt M, Keisari Y, Kelson I (2007) Treatment of solid tumors by interstitial release of recoiling short-lived alpha emitters. Phys Med Biol 52:5025–5042. 10.1088/0031-9155/52/16/021 17671351 10.1088/0031-9155/52/16/021

[17] Cooks T, Arazi L, Schmidt M, Marshak G, Kelson I, Keisari Y (2008) Growth retardation and destruction of experimental squamous cell carcinoma by interstitial radioactive wires releasing diffusing alpha-emitting atoms. Int J Cancer 122:1657–1664. 10.1002/ijc.23268 18059026 10.1002/ijc.23268

[18] Cooks T, Schmidt M, Bittan H, Lazarov E, Arazi L, Kelson I, Keisari Y (2009) Local control of lung derived tumors by diffusing alpha-emitting atoms released from intratumoral wires loaded with radium-224. Int J Radiat Oncol Biol Phys 74:966–973. 10.1016/j.ijrobp.2009.02.063 19480976 10.1016/j.ijrobp.2009.02.063

[19] Cooks T, Arazi L, Efrati M, Schmidt M, Marshak G, Kelson I, Keisari Y (2009) Interstitial wires releasing diffusing alpha emitters combined with chemotherapy improved local tumor control and survival in squamous cell carcinoma-bearing mice. Cancer 115:1791–1801. 10.1002/cncr.24191 19197995 10.1002/cncr.24191

[20] Cooks T, Tal M, Raab S, Efrati M, Reitkopf S, Lazarov E, Etzyoni R, Schmidt M, Arazi L, Kelson I, Keisari Y (2012) Intratumoral 224Ra-loaded wires spread alpha-emitters inside solid human tumors in athymic mice achieving tumor control. Anticancer Res 32:5315–5321 23225432

[21] Horev-Drori G, Cooks T, Bittan H, Lazarov E, Schmidt M, Arazi L, Efrati M, Kelson I, Keisari Y (2012) Local control of experimental malignant pancreatic tumors by treatment with a combination of chemotherapy and intratumoral 224radium-loaded wires releasing alpha-emitting atoms. Transl Res 159:32–41. 10.1016/j.trsl.2011.08.009 22153808 10.1016/j.trsl.2011.08.009

[22] Keisari Y, Hochman I, Confino H, Korenstein R, Kelson I (2014) Activation of local and systemic anti-tumor immune responses by ablation of solid tumors with intratumoral electrochemical or alpha radiation treatments. Cancer Immunol Immunother 63:1–9. 10.1007/s00262-013-1462-2 23955682 10.1007/s00262-013-1462-2 PMC11029492

[23] Confino H, Schmidt M, Efrati M, Hochman I, Umansky V, Kelson I, Keisari Y (2016) Inhibition of mouse breast adenocarcinoma growth by ablation with intratumoral alpha-irradiation combined with inhibitors of immunosuppression and CpG. Cancer Immunol Immunother 65:1149–1158. 10.1007/s00262-016-1878-6 27495172 10.1007/s00262-016-1878-6 PMC11028980

[24] Popovtzer A, Rosenfeld E, Mizrachi A, Bellia SR, Ben-Hur R, Feliciani G, Sarnelli A, Arazi L, Deutsch L, Kelson I, Keisari Y (2019) Initial Safety and Tumor Control Results from a first-in-human multicenter prospective trial evaluating a Novel Alpha-Emitting Radionuclide for the treatment of locally advanced recurrent squamous cell carcinomas of the skin and Head and Neck. Int J Radiat Oncol Biol Phys. 10.1016/j.ijrobp.2019.10.048 31759075 10.1016/j.ijrobp.2019.10.048

[25] D’Andrea MA, VanderWalde NA, Ballo MT, Patra P, Cohen GN, Damato AL, Barker CA (2023) Feasibility and safety of Diffusing Alpha-Emitter Radiation Therapy for recurrent or unresectable skin cancers. JAMA Netw Open 6:e2312824. 10.1001/jamanetworkopen.2023.12824 37166798 10.1001/jamanetworkopen.2023.12824 PMC10176125

[26] Heger G, Roy A, Dumancic M, Arazi L (2023) Alpha dose modeling in diffusing alpha-emitters radiation therapy-part I: single-seed calculations in one and two dimensions. Med Phys 50:1793–1811. 10.1002/mp.16145 36464914 10.1002/mp.16145

[27] Heger G, Dumancic M, Roy A, Arazi L (2023) Alpha dose modeling in diffusing alpha-emitters radiation therapy. Part II: lattice studies. Med Phys 50:1812–1823. 10.1002/mp.16155 36517936 10.1002/mp.16155

[28] Arazi L, Cooks T, Schmidt M, Keisari Y, Kelson I (2010) The treatment of solid tumors by alpha emitters released from (224)Ra-loaded sources-internal dosimetry analysis. Phys Med Biol 55:1203–1218. 10.1088/0031-9155/55/4/020 20124656 10.1088/0031-9155/55/4/020

[29] Reardon DA, Brandes AA, Omuro A, Mulholland P, Lim M, Wick A, Baehring J, Ahluwalia MS, Roth P, Bahr O, Phuphanich S, Sepulveda JM, De Souza P, Sahebjam S, Carleton M, Tatsuoka K, Taitt C, Zwirtes R, Sampson J, Weller M (2020) Effect of Nivolumab vs Bevacizumab in patients with recurrent glioblastoma: the CheckMate 143 phase 3 Randomized Clinical Trial. JAMA Oncol 6:1003–1010. 10.1001/jamaoncol.2020.1024 32437507 10.1001/jamaoncol.2020.1024 PMC7243167

