# Supplementary material for: Stereotactic implantation of diffusing alpha-emitters radiation therapy sources in the swine brain: a potential new focal therapy for brain tumors
Source: J Neurooncol. 2025 Jan 2;172(2):387–96. doi: 10.1007/s11060-024-04919-5 (PMC11937107; doi:10.1007/s11060-024-04919-5)
Supplement: Supplementary file 1 — Supplementary Material 1 [file 11060_2024_4919_MOESM1_ESM.docx]

# **Stereotactic Implantation of Diffusing Alpha-emitters Radiation Therapy Sources in the Swine Brain: a Potential New Focal Therapy for Brain Tumors**

**Journal of neuro-oncology**

Yigal Shoshan MD ^1^, Moshe J Gomori MD ^2^, Lior Moss DVM ^3^, Saleem Eben Bari DVM ^3^, Nir Edery DVM ^3^, Robert B. Den MD ^4^, Lior Arazi PhD ^5^, Aron Popovtzer MD  ^6^, Jon Feldman PhD ^6^_,_ Samuel Moscovici MD^1^

^1^Department of Neurosurgery, Hadassah-Hebrew University Medical Center, Jerusalem, Israel.

^2^Department of Radiology, Hadassah-Hebrew University Medical Center, Jerusalem, Israel

^3^Department of Pathology, Kimron Veterinary Institute, Bet Dagan, Israel

^4^ Alpha Tau Medical, Jerusalem, Israel.

^5^Unit of Nuclear Engineering, Faculty of Engineering Sciences, Ben-Gurion University of the Negev, Be'er-Sheva, Israel.
^6^ Sharett Institute of Oncology, Hebrew University-Hadassah Medical Center, Jerusalem, Israel.

**Corresponding Author**: E-mail: yigalshoshan@mac.com.

**Supplementary Information**

**Animal acclimation and monitoring**

The animals were acclimated for at least 5 days before Alpha-DaRT implantation, were housed within a limited-access Large Animal unit in pre-disinfected pens, were provided with commercial swine diet and were allowed free access to drinking water supplied by automated watering valves. Animals were monitored twice daily by the veterinary staff for the whole study duration, unless mentioned otherwise.

**Sedation, Analgesia, or Anesthesia**

For premedication, xylazine 10%, 2 mg/kg (Anased, AKORN, USA) + ketamine 10%, 10 mg/kg (Clorkeam, Vetoquinol, France) were given by i.m. injection. Induction was performed using 3% isoflurane with 100% oxygen via a mask (Isoflurane, USP Terrell, Piramal Critical Care, USA). Intubation and maintenance on isoflurane were performed in the concentration of 1-3% with 100% oxygen (Isoflurane, USP Terrell, Piramal Critical Care, USA). Buprenorphine (vetmarket ltd, Israel) 0.05-0.1 mg/kg was given by i.m. injection, twice a day for 2-4 days (vet decision as needed).

**Radial applicator usage**

Before operation, the rotation mechanism was first connected to the working channel, and the 16 G tool was placed in the brain biopsy needle. Then, the required length of the needle was adjusted according to the treatment plan and was fixed in place by a screw. Next, the brain biopsy needle with the 16 G tool was inserted into the rotational mechanism. Immediately prior to deployment, the applicator was washed with saline, and the stylet of the radial handle was inserted from the upper side to locate the sources at the required location. The limiter and cartridge with the radial handle were assembled into the biopsy needle. The sources were deployed by pushing the handle and then, for each source the rotation mechanism was rotated to the next position (1-7). After deploying all 7 sources, the cartridge was removed from the biopsy needle. For the deployment of an additional layer, a layer index was changed at the rotational mechanism to the next, and the radial handle was reset.

**Intraoperative Fluoroscopy, postoperative computed tomography (CT), and magnetic resonance imaging (MRI)**

Prior to animal studies, an inert titanium-based Alpha-DaRT source was inserted into a liquid 0.3% agar that was then allowed to solidify. The fixed source was imaged successfully using the three sequences (T1W, T2W, and FLAIR) commonly used for brain tumor patients (Fig. S2). In vivo, all imaging procedures were performed while under anesthesia (Fig. 1). Fluoroscopy images (FD20 or Zenition 70, Philips, Netherlands) of the head were acquired during surgery to verify needle tip location before source deployment, based on the neurosurgeon’s decision. An MRI scan of the brain (Voyager 1.5 T, GE Healthcare, United States) was performed using a protocol similar to that used for brain tumor patients on day 30 ± 7 and 60 ± 7 post insertion, to evaluate potential damage to brain tissue in and around the treated area. We looked for hemorrhage, edema, surgical site infection, inflammatory response, and implant shape and movement. A pre-op CT scan (Brilliance64, Philips, Netherlands) for treatment planning and design of the 3D-printed implantation template was performed. Additional seven CT scans of the head were done to verify that the Alpha-DaRT sources did not migrate outside the brain or move inside the brain. These scans were performed, unless mentioned otherwise, on day 0 immediately after the source insertion, and on days 3, 7, 14, 21, 31, and termination day, during which every swine was placed at the same position as on the CT scan of day 0.

**MRI and CT parameters**

MRI parameters were as follows: 3D T1-CUBE slice thickness = 1 mm, TR =552 ms, TE = 15.22 ms, gap = 0, Flip angle = 90. 3D-FSGR slice thickness = 1.1 mm TR = 17.4 ms, TE= 2.84 ms, gap=0, Flip angle 20. 3D-FLAIR slice thickness =1.2 mm, TR = 8602 s, Te = 147.61 ms. 2D-T2TSE slice thickness = 2mm, TR= 4399 ms, TE = 102.43 ms, gap = 2.4 mm, Flip angle = 160. 2D-DWI slice thickness = 2.1mm, TR = 7791 ms, gap 2.5 mm, Flip angle 90. 2DSWAN TR = 65.9 ms, TE = 48.15 ms, gap = 1 mm, Flip angle 25). CT parameters were as follows: slice width=0.8 mm; increment=0.4 mm; voltage=120 kV; milliamperage second=587 mAs; current=459 mA; time factor=1279 ms.

**Animal clinical and neurological examination**

The animals underwent a clinical and neurological examination once a week (Fig. 1) starting one day after the procedure. The clinical examination included monitoring body temperature, heart rate, respiratory rate, appetite, incision line condition, and behavior. The neurological examination included monitoring the swine’s mental status, head posture, body posture, gait, postural reactions, and cranial nerve function. During the first week after the procedure the swine’s appetite, gait, and behavior were monitored by the veterinarian staff. After the first week the swine were transferred to their standard pen and examined twice daily.

**Cerebrospinal fluid** **(CSF) and blood tests**

Cerebrospinal fluid (CSF) samples were collected under general anesthesia on day 0 (before source implantation) and on days 7-8, 14-15, and termination day unless mentioned otherwise (Fig. 1). Samples were obtained through lumbar puncture and collected in a non-additive tube. The sampling area was shaved and disinfected. A sterile spinal needle was inserted into the lumbar cistern between L4 and L5 vertebrae. One swine in phase III was sacrificed due to complications of multiple attempts at a spinal tap that led to rectal prolapse and perforation. Thereafter the number of CSF sampling trials was limited to three CSF punctures. Blood samples were taken on days 0, 2-3, 7-8, 14-15, 21, 30-31, and on termination day. No baseline sample on day 0 or biochemistry tests were performed for the inert phase. The samples were obtained under general anesthesia from the jugular vein using a sterile needle.

**CSF and Blood sample processing**

1 ml of CSF fluid was collected in a sodium fluoride tube for lactate level analysis. 1.5 ml of CSF fluid was collected in an ACD-A tube for Glucose and total protein analysis. The samples were kept in ice for 1.5 hours until analyzed using a Cobas 6000 spectrophotometer with a chamber temperature of 37 degrees Celsius.

The blood was drawn into ACD-A tubes for biochemistry analysis. Thirty minutes after sampling, the tubes were centrifuged at 3.5 RPM for 10 minutes. Biochemistry analysis was conducted using a Cobas 6000 spectrophotometer. Blood was drawn into EDTA anticoagulant tubes for CBC analysis. The samples were kept cool (at 2-8 degrees Celsius). The CBC analysis was performed using an Advia 2120 hematology analyzer system. Both CBC and biochemistry analyses were completed within 72 hours of sampling.

**^212^Pb Measurements and external dose-rate measurements**

For swine in the active sources’ phases, urine, blood, feces, and cerebrospinal fluid samples (2-2.5 ml each) were collected for ^212^Pb radioactivity measurements on day 0 (baseline, before source implantation), day 7-8, and day 14-15 (Fig. 1). Blood and CSF were collected as described above and urine samples were obtained under general anesthesia via ultrasound-guided cystocentesis using a sterile needle and syringe and collected in a non-additive tube. External dose-rate measurements around the swine’s heads were done on days 0 (baseline pre-insertion + post-insertion), 3, 5, 7, and 10 (only for swine in the active phases). ^212^Pb activity was determined using a well-type NaI(Tl) gamma counter (Automatic Gamma Counter, Hidex), focusing on the 239 keV photopeak.

**Pathology and histopathology**

Gross morphology was performed following euthanasia. Whole brain specimens were collected and placed in 10% formalin solution for at least 14 days. The preserved specimens were embedded in paraffin. Histological sections (5 or 10 μm) were prepared, stained with hematoxylin-eosin (H&E), and analyzed for tissue damage by a pathologist.

**Absorbed dose estimates**

Analysis of local therapeutic dose was performed per each swine (Table S2, Figure S5) using the MIM Symphony treatment planning software (V7.3.0), implementing the Alpha-DaRT diffusion-leakage model with low-LET contributions [1, 2]. The procedure consisted of positioning sources in 3D, based on the applicator penetration path and source deployment geometry, and calculating the dose in all voxels comprising the relevant volume around the sources using the dose model. As shown in [2, 3] both the alpha dose and the low-LET dose drop to < 1 Gy at a distance of ~4 mm from the outermost source, ensuring the sparing of surrounding healthy tissue outside of the treatment region.

A gross estimate for the systemic alpha-particle absorbed dose to the swine brain (due to ^212^Pb leakage from the treated region through the blood) can be done as follows. In humans, the brain holds 1.2% of the total blood volume [4]. Since the average brain mass in adult human females is 1.3 kg, and the total blood volume is 3.9 liters [4], the blood volume per brain tissue is 0.036 ml/g. We assume the same ratio holds in swine. The average measured normalized specific ^212^Pb activity in blood in this work is 0.14 Bq/ml per µCi ^224^Ra in secular equilibrium (with the ^224^Ra activity calculated for the time of blood sample collection). With 0.036 ml blood per gram of brain tissue, the normalized specific ^212^Pb activity in the brain in secular equilibrium is therefore 0.036 ml/g × 0.14 Bq/ml/µCi = 5.0×10^-3^ Bq/g/µCi. We assume, for simplicity, that this ratio holds for the entire duration of the treatment, and that the energy of the alpha particle emitted by either ^212^Bi or ^212^Po (with a weighted average of 7.8 MeV = 1.25×10^-12^ J) is fully deposited in brain tissue. Under these assumptions, the systemic absorbed alpha dose rate (due to alpha decays in the blood) throughout the treatment is 2.3 ×10^-8^ Gy/h/µCi. For an initial ^224^Ra activity of 126 µCi (the maximal activity in this study), the initial systemic effective absorbed rate (neglecting the initial buildup of ^212^Pb) is therefore 2.9 ×10^-6^ Gy/h. The total systemic absorbed dose is approximately the initial effective dose rate multiplied by the mean lifetime of ^224^Ra (125.7 h), giving 3.6 ×10^-4^ Gy. According to calculations done using the Alpha-DaRT biokinetic model [5] the blood contributes 73% of the total systemic alpha dose to the brain (the rest of the dose is due to uptake of ^212^Pb by the brain tissue itself). Up-to-date unpublished calculations using the latest ICRP models indicate that the blood contributes 78-98% of the total systemic alpha dose to the brain. With this, the total systemic alpha dose to the brain for 126 µCi ^224^Ra is (4.1±0.5) ×10^-4^ Gy. Note that this calculation assumes no contribution to the systemic absorbed alpha dose by the CSF, since it is contained in macroscopic volumes (cranial and spinal subarachnoid spaces and ventricles), much larger than the range of alpha particles. As shown in [5], the summed beta and gamma physical dose to the brain is ~5 times smaller than the absorbed dose by alpha particles. Considering the high relative biological effectiveness (RBE) of alpha particles (with a nominal value of 5 for deterministic effects, as recommended by MIRD Pamphlet 22 [6]), the combined beta and gamma contribution to the RBE-weighted absorbed dose drops to < 4%. If one assumes an alpha tolerance dose of 12 Gy [5], the absorbed dose to normal brain tissue due to ^212^Pb leakage through the blood for a treatment consisting of 126 µCi ^224^Ra is ~30,000 smaller than the tolerance.

As noted above, both the alpha dose and beta dose drop below 1 Gy at a distance of ~4 mm from the outermost sources. Additional calculations show that for the total ^224^Ra activities applied in this study the beta and gamma dose is already smaller than ~0.001 Gy ~10 mm away from the treated region, becoming smaller than the systemic dose at ~11 mm.

**Spatial-temporal source location analysis**

To analyze possible source movement in the brain tissue, we compared the location of each source between two different time points: immediately after source insertion and close to termination. The analysis was based on CT DICOM images, imported into the treatment planning software (MIM Symphony V7.3.0), by manually extracting the coordinates of the source ends. The movement of individual Alpha-DaRT sources during the follow-up period was assessed according to the following procedure: Post-implantation-CT-scan (on day 0) and last follow-up-CT-scan images were registered as previously described [7] and matched between sources, so that each pair of sources corresponded to the same actual source at different times [3]. The minimal convex shape that encapsulates the sources together with their respective total error (see below) was defined as the “3D source enclosure” and was calculated according to the source locations on the day-0 CT. Sources that were outside of the 3D source enclosure on the last follow-up scan were labeled as “outliers”. The number of outliers and the maximal distance from the 3D source enclosure to each source were calculated to express the movement of the sources.

**Total error estimates**

The registration error (i.e., the spatial error in matching the anatomical contours in three dimensions) was estimated as ~3.5 mm, much larger than the uncertainty in identifying the source edge coordinates (~1 mm). The total error related to source location was estimated by adding these two uncertainty components in quadrature, giving 3.6 mm.

**References**

1. Arazi L (2020) Diffusing alpha-emitters radiation therapy: approximate modeling of the macroscopic alpha particle dose of a point source. Phys Med Biol 65: 015015 doi:10.1088/1361-6560/ab5b73

2. Epstein L, Heger G, Roy A, Gannot I, Kelson I, Arazi L (2024) The low-LET radiation contribution to the tumor dose in diffusing alpha-emitters radiation therapy. Med Phys 51: 3020-3033 doi:10.1002/mp.16885

3. Heger G, Roy A, Dumancic M, Arazi L (2023) Alpha dose modeling in diffusing alpha-emitters radiation therapy-Part I: single-seed calculations in one and two dimensions. Med Phys 50: 1793-1811 doi:10.1002/mp.16145

4. (2002) Basic anatomical and physiological data for use in radiological protection: reference values. A report of age- and gender-related differences in the anatomical and physiological characteristics of reference individuals. ICRP Publication 89. Ann ICRP 32: 5-265

5. Arazi L, Cooks T, Schmidt M, Keisari Y, Kelson I (2010) The treatment of solid tumors by alpha emitters released from (224)Ra-loaded sources-internal dosimetry analysis. Phys Med Biol 55: 1203-1218 doi:10.1088/0031-9155/55/4/020

6. Sgouros G, Roeske JC, McDevitt MR, Palm S, Allen BJ, Fisher DR, Brill AB, Song H, Howell RW, Akabani G, Committee SM, Bolch WE, Brill AB, Fisher DR, Howell RW, Meredith RF, Sgouros G, Wessels BW, Zanzonico PB (2010) MIRD Pamphlet No. 22 (abridged): radiobiology and dosimetry of alpha-particle emitters for targeted radionuclide therapy. J Nucl Med 51: 311-328 doi:10.2967/jnumed.108.058651

7. Sadoughi A, Sarkar A, Novotney C, Chan C, Moreno G, Oren S, Moss L, Aharoni K, Bari SE, Edery N, Ohri N (2024) Bronchoscopic deployment and implantation of Diffusing alpha-emitters Radiation Therapy into the lung and mediastinum for treatment of lung cancer: a pre-clinical safety and feasibility study. Transl Lung Cancer Res 13: 60-75 doi:10.21037/tlcr-23-639

**Supplementary Tables and Figures**

**Table S1. Number of sources implanted in each hemisphere**

| Phase | Swine # | Activity | Termination  day | Hemisphere | # of layers | Deepest layer | Deep layer | Superficial layer | **# of sources** | **Total # sources** |
| --- | --- | --- | --- | --- | --- | --- | --- | --- | --- | --- |
| I | DP-13176 | Inert | 66 | Right | 3 | 7 | 7 | 7 | **21** | **21** |
|  | DP-13177 | Inert | 60 | Left | 3 | 7 | 7 | 7 | **21** | **21** |
| II | DP-13253 | Active | 59 | Right | 2 |  | 7 | 7 | **14** | **28** |
|  | DP-13253 |  |  | Left | 2 |  | 7 | 7 | **14** |  |
|  | DP-13254 | Active | 59 | Right | 3 | 7 | 7 | 7 | **21** | **42*** |
|  | DP-13254 |  |  | Left | 3 | 7 | 7 | 7 | **21** |  |
| III | DP-13331 | Active | 1 | Right | 2 |  | 7 | 7 | **14** | **28**** |
|  | DP-13331 |  |  | Left | 2 |  | 7 | 7 | **14** |  |
|  | DP-13332 | Active | 62 | Right | 2 |  | 7 | 7 | **14** | **28** |
|  | DP-13332 |  |  | Left | 2 |  | 7 | 7 | **14** |  |
| IV | DP-13577 | Active | 90 | Right | 2 |  | 7 | 6*** | **13** | **27** |
|  | DP-13577 |  |  | Left | 2 |  | 7 | 7 | **14** |  |
|  | DP-13578 | Active | 90 | Right | 1 |  |  | 7 | **7** | **11** |
|  | DP-13578 |  |  | Left | 1 |  |  | 4 | **4** |  |

***** Mild temporary neurological signs. ****** Sacrificed due to damage to the spinal cord during CSF sampling that resulted in a lack of anal tonus and rectal prolapse, the next day perforation in the intestines. *** one source was not inserted properly due to proximity to the skull

**Table S2. Dosimetry** **calculation**. Total ^224^Ra activity and the therapeutic volume in which the dose is larger than the prescribed dose. The volume was calculated using MIM Symphony V7.3.0, implementing the Alpha-DaRT diffusion-leakage model including low-LET emissions [1, 2] with 100 Gy used as the prescribed dose (RBE-weighted alpha dose + low-LET dose, assuming RBE=5).

| **Study** | **Animal** | **Total Activity [μCi]** | **Therapeutic Volume [ml]** |
| --- | --- | --- | --- |
| Active 1 | DP13253 | 84 | 3.06 |
|  | DP13254 | 126 | 3.28 |
| Active 2 | DP13331 | 84 | 3.16 |
|  | DP13332 | 84 | 2.49 |
| Active 3 | DP13444 | 84 | 3.16 |
|  | DP13445 | 84 | 3.01 |
| Active 4 | DP13577 | 81 | 2.89 |
|  | DP13578 | 39 | 1.34 |


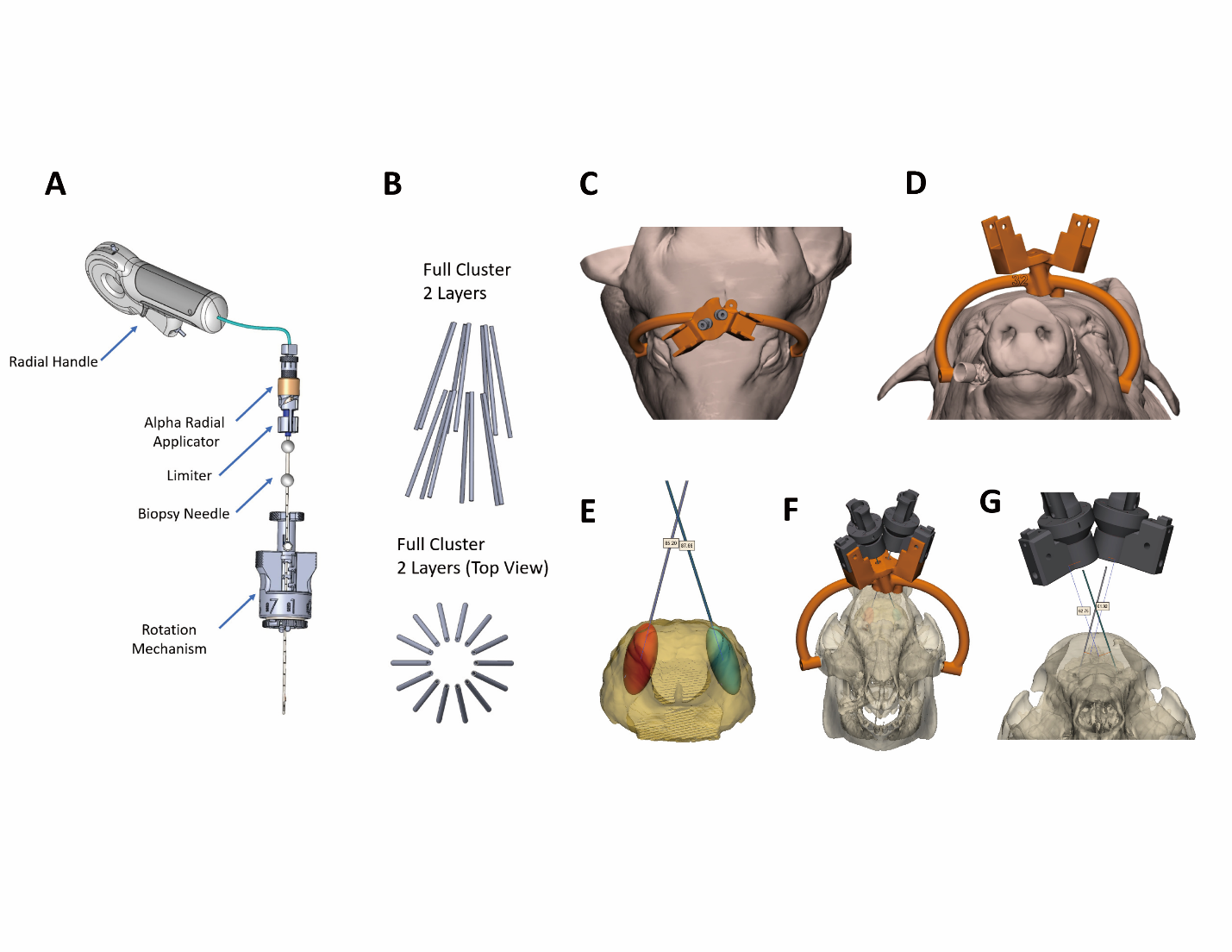
**Figure S1. Alpha-DaRT radial applicator, sources cluster configuration, and procedure plan**. **A)** The Alpha-DaRT radial applicator is composed of a cartridge that contains 7 sources; a 16 G tool that guides the cartridge at the precise angle by the slope in its edge; A handle that its stylet pushes the source from the cartridge into tissue (in the same length as the source); a rotation mechanism that aims the whole biopsy needle into the exact angle corresponding to its axis. **B)** an umbrella-like configuration of 7 sources cluster in a layer. **C)** Dorsal view of the swine head, the template-frame (Orange), and two reducing tubes (Grey). **D)** Frontal view of the swine’s head with the template-frame (orange). **E)** Frontal view of the swine’s skull. The blue and purple lines denote the drill burrs. The red and blue ellipses denote the area of source deployment in parietal lobe of the swine’s brain. **F)** Frontal view of the pig’s skull with the template frame (orange), the rotation mechanism (Black) and the drill burrs. **G)** Frontal view of the swine’s skull with the rotation mechanism (Black) and the drill burrs, without the template-frame.


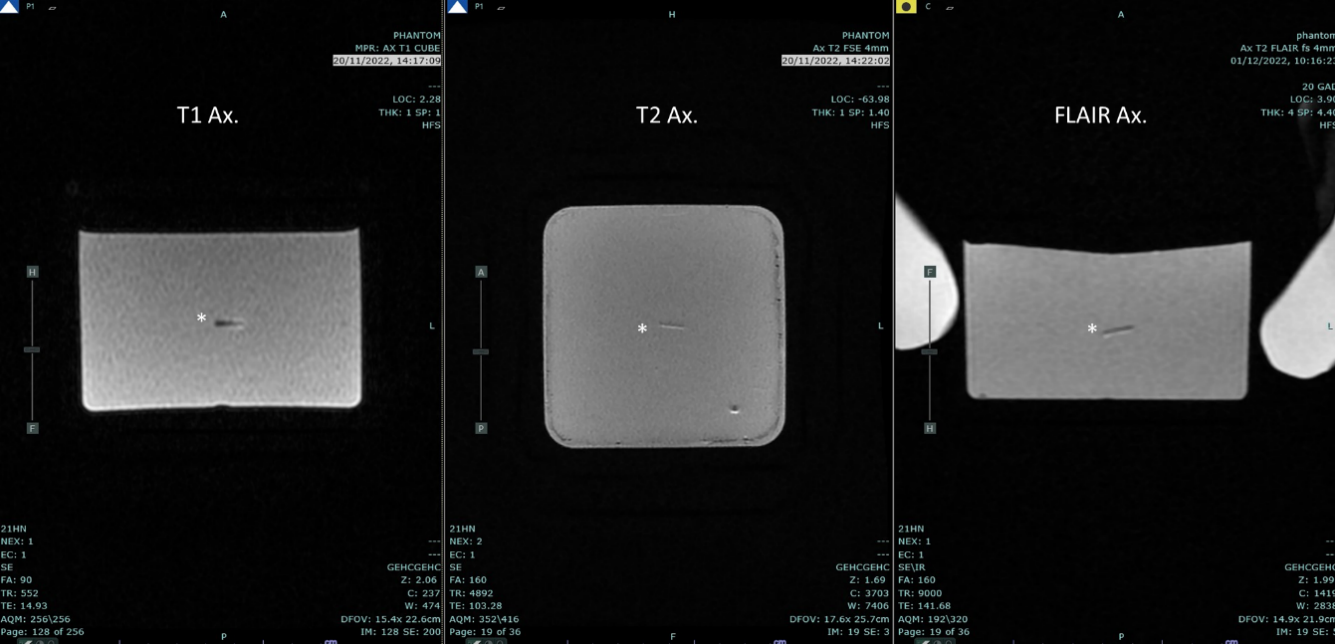


**Figure S2.** **MR Image of an Alpha-DaRT source in a phantom model.** MRI data sets with three common sequences (T1W, T2W, and) of 0.3% agar media phantom containing a titanium source. Note - there are no image artifacts. White asterisk - titanium source.


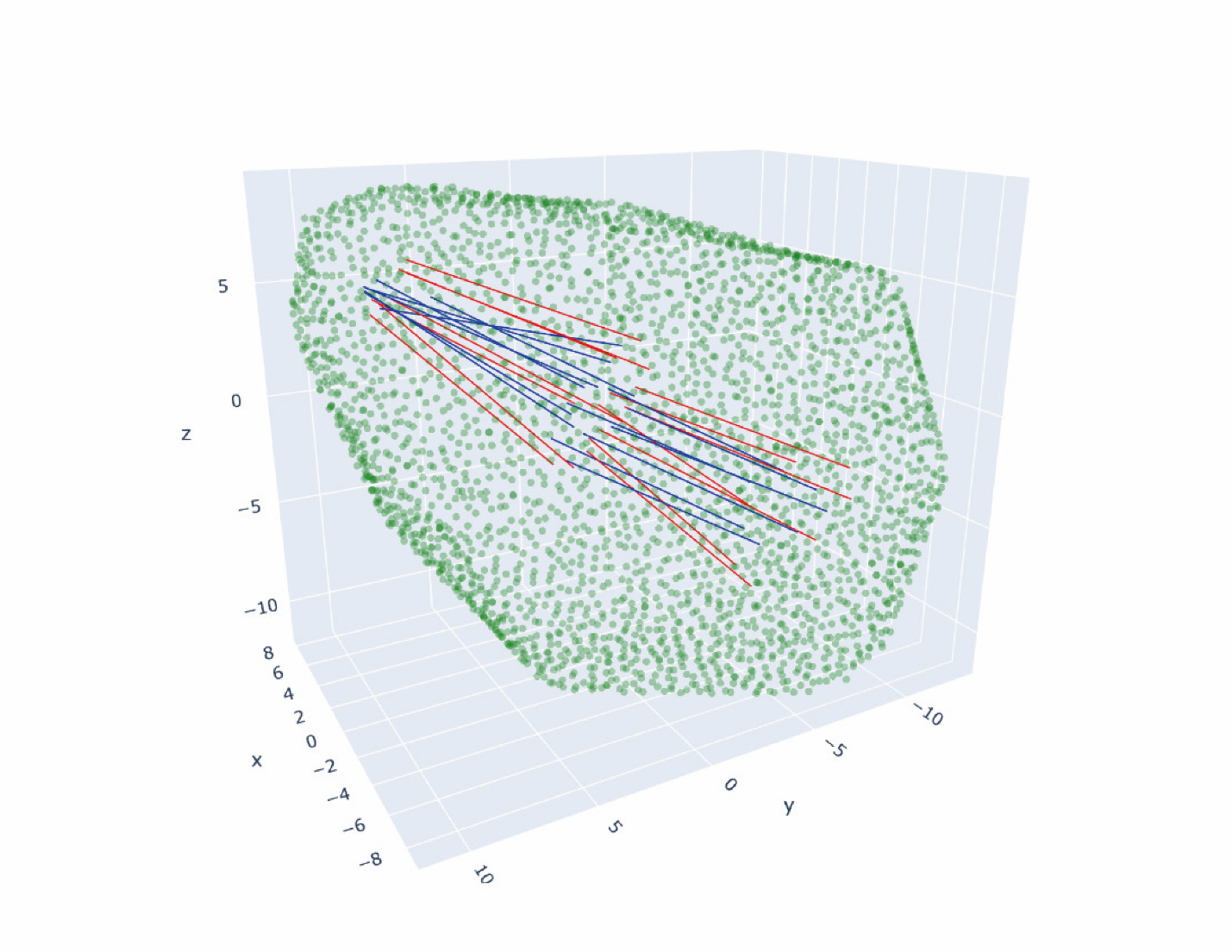
**Figure S3. Spatial-temporal localization analysis.** a graphical example of sources movement in a sources cluster of swine 13332 in space and time. Red lines represent the positions of the sources on day 0. Blue lines represent the positions of the sources on day 59. The 3D source enclosure (green) is defined as the minimal shape that encapsulates the sources plus the total error (registration + source coordinate identification errors) and was used to identify outliers (sources that are partially or totally out of the 3D source enclosure). Axis units are millimeters.


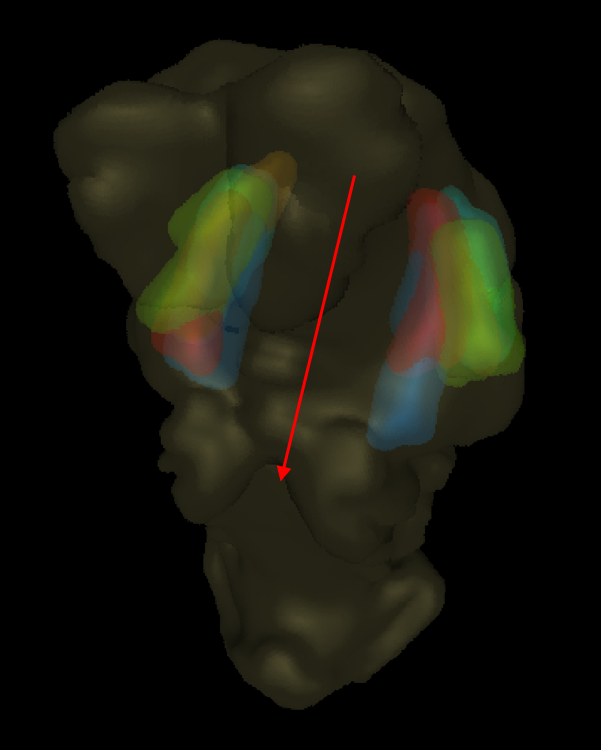


**Figure S4.** **Animal DP13254 implanted sources along with their volumetric dose (Alpha dose >20 Gy) representation relative to the rest of the animals**. The animal received 3 layers of umbrella-like configuration of 7-sources. The brain contour of animal DP13332 (faded olive), together with the volumetric dose representation of the sources of DP13332 (green contour), DP13577 (cyan contour), DP13578 (brown contour), DP13253 (red contour) and DP13254, in which part of the sources were implanted deeper relatively to the other animals (blue contour) in the left hemisphere. The inferior direction (red arrow) indicates that these sources were implanted deeper compared to the others, closer to the brain stem.


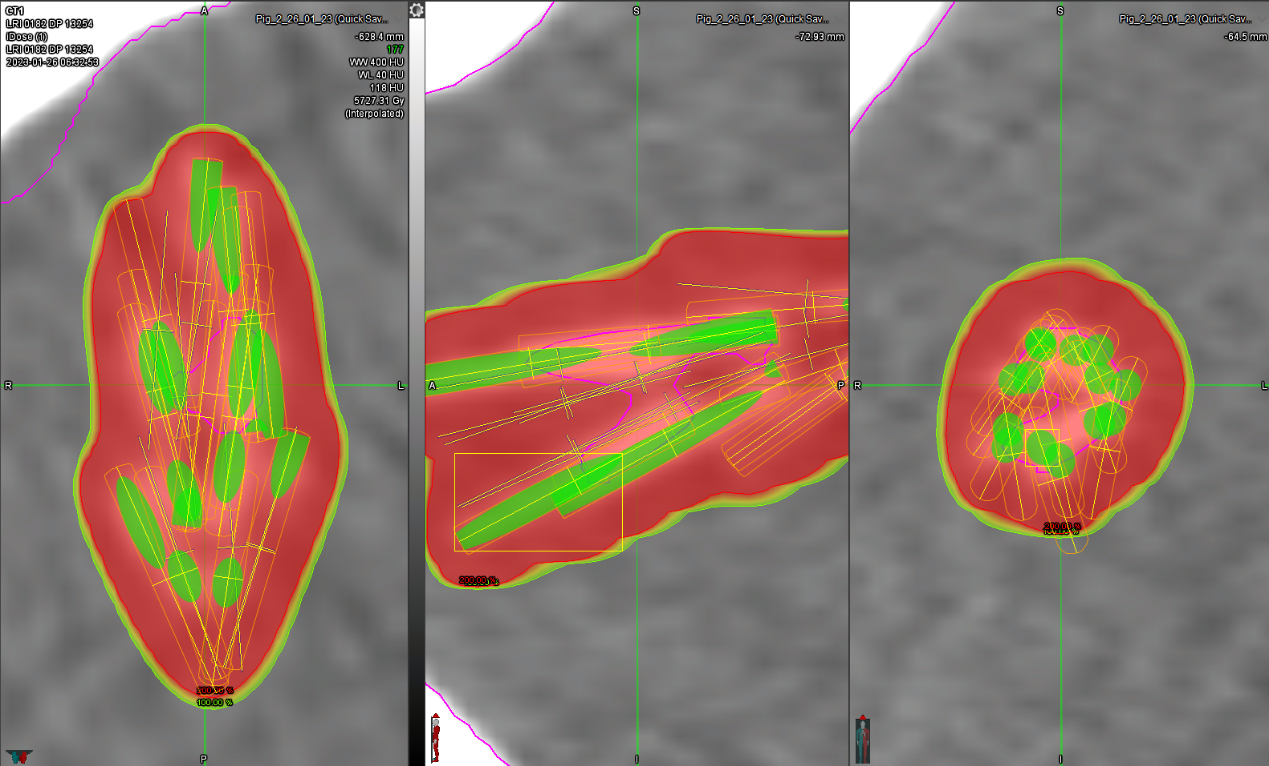


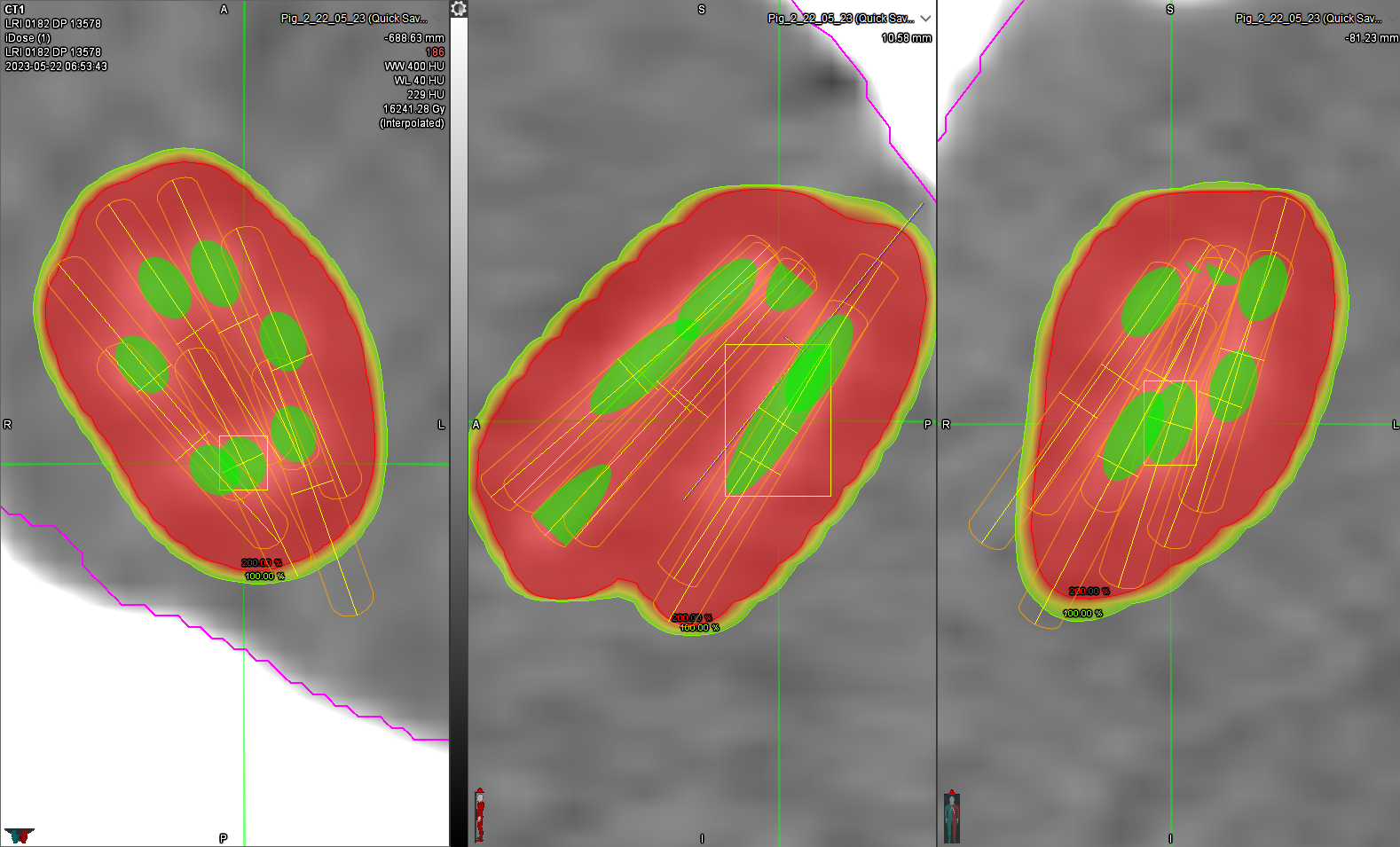


**Figure S5. Representative dose distributions** (with color-washed area) **in two swine:** screenshots from MIM Symphony (upper panel) showing a CT image of a swine that received the maximal activity (126 µCi ^224^Ra) with a therapeutic volume (>100 Gy of RBE-weighted total dose) of 3.28 mL; Lower panel: a swine that received the minimal activity (39 µCi ^224^Ra), for which the therapeutic volume is 1.34 mL. Green-colored dose regions denote areas of >100% of the prescribed dose and red of >200% of the prescribed dose. The yellow line represents the source outlines in 3D and green ellipses their cross section at the image plane.
